# Hepatocellular carcinoma: pathogenesis, molecular mechanisms, and treatment advances

**DOI:** 10.3389/fonc.2025.1526206

**Published:** 2025-04-08

**Authors:** Zhixian Ding, Lusheng Wang, Jiting Sun, Lijie Zheng, Yu Tang, Heng Tang

**Affiliations:** ^1^ General Clinical Research Center, Wanbei Coal-Electricity Group General Hospital, Suzhou, China; ^2^ Laboratory of Inflammation and Repair of Liver Injury and Tumor Immunity, Wanbei Coal-Electricity Group General Hospital, Hefei, China

**Keywords:** hepatocellular carcinoma, immune-targeted therapy, molecular signaling pathways, macrophages, cirrhosis

## Abstract

Hepatocellular Carcinoma (HCC), a highly prevalent malignancy, poses a significant global health challenge. Its pathogenesis is intricate and multifactorial, involving a complex interplay of environmental and genetic factors. Viral hepatitis, excessive alcohol consumption, and cirrhosis are known to significantly elevate the risk of developing HCC. The underlying biological processes driving HCC are equally complex, encompassing aberrant activation of molecular signaling pathways, dysregulation of hepatocellular differentiation and angiogenesis, and immune dysfunction. This review delves into the multifaceted nature of HCC, exploring its etiology and the intricate molecular signaling pathways involved in its development. We examine the role of immune dysregulation in HCC progression and discuss the potential of emerging therapeutic strategies, including immune-targeted therapy and tumor-associated macrophage interventions. Additionally, we explore the potential of traditional Chinese medicine (TCM) monomers in inhibiting tumor growth. By elucidating the complex interplay of factors contributing to HCC, this review aims to provide a comprehensive understanding of the disease and highlight promising avenues for future research and therapeutic development.

## 1 Introduction

Liver diseases cause over 2 million deaths annually, accounting for 4% of all global deaths (1 in every 25 deaths) (1). Currently, liver diseases are the 11th leading cause of death, but liver-related mortality may have been underestimated (2).

Approximately two-thirds of all liver-related deaths occur in men (1, 3). The main causes of death are complications of cirrhosis and HCC. Liver diseases are diverse, including hepatitis, alcoholic liver disease, metabolic dysfunction-associated fatty liver disease(MAFLD), cirrhosis, and liver cancer. These diseases share common features such as varying degrees of inflammation and liver cell damage (4–6). In the early stages, symptoms may not be significant, but long-term accumulation can lead to cirrhosis or even liver cancer (HCC), ultimately resulting in death (7, 8).

**Figure 1:**
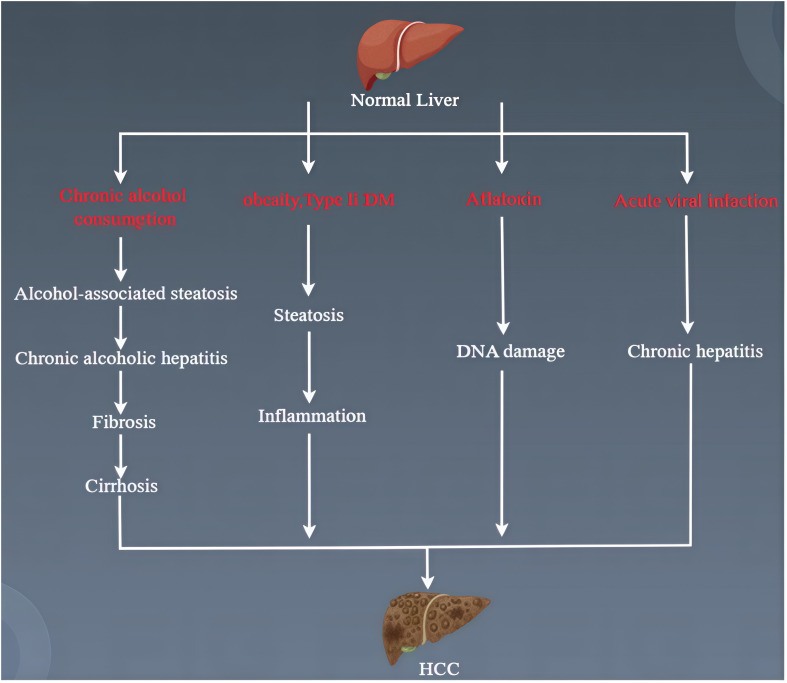
Course of HCC pathogenesis.

HCC develops through a complex, multi-stage biological process. MAFLD, alcoholic liver disease, autoimmune hepatitis, hepatitis B, and hepatitis C (**Figure 1**) are all potential causes of HCC (9). Currently, the progression of HCC is often accompanied by genetic and epigenetic modifications, oxidative stress, inflammation, and immune involvement (10). Liver cancer stem cells (LCSC) play a critical role in cancer occurrence, metastasis, recurrence, and treatment resistance, affecting the dedifferentiation of mature hepatocytes and bile duct cells (11, 12). The loss of tumor suppressor proteins p53/p21 leads to the dedifferentiation of mature liver cells into progenitor-like cells, which further develop into HCC with gene mutations in the Wnt and Notch signaling pathways (12). The insulin-like growth factor (IGF) signaling pathway is involved in the occurrence, progression, and metastasis of HCC (13). Moreover, signaling pathways controlling growth factor receptors (such as FGFR, TGFA, EGFR, and IGFR), cytoplasmic intermediates (such as PI3K-AKT-mTOR, RAF/ERK/MAPK), and key cell differentiation pathways (such as Wnt-catenin, JAK/STAT, Hippo, Hedgehog, and Notch) all influence the progression of HCC (14). In recent years, the greatest focus in cancer treatment has been on tumor-associated macrophages and liver cancer immunotherapy.

This review will provide a comprehensive understanding of the pathogenesis of liver diseases, current research advancements in cancer treatment, and future perspectives, offering clinical researchers a theoretical foundation and reference for potential therapeutic targets in treating liver cancer patients.

## 2 Pathogenesis of HCC

### 2.1 Alcoholic liver disease

Currently, 43% of the global population consumes alcohol, and excessive drinking is a significant risk factor for disease and death worldwide (15, 16). It is reported that alcohol increases the mortality rate related to liver disease by 260 times, cardiovascular disease by 3.2 times, and cancer by 5.1 times (17). About 35% of patients with alcohol use disorder (AUD) develop various forms of ALD (18). ALD often coexists with viral hepatitis and MAFLD. However, compared to liver diseases of other causes, alcoholic liver disease is more likely to progress to cirrhosis. Additionally, with the rising global prevalence of obesity and type 2 diabetes, alcohol-related liver damage is increasing. Obesity and metabolic syndrome can synergistically exacerbate the severity of ALD at all stages (19).

Drinking alcohol also increases the risk of liver cancer for those who are overweight, obese, or have liver cirrhosis associated with MAFLD (20, 21). Without global interventions, ALD-related mortality is expected to rise significantly.

Alcohol metabolism in the body occurs through oxidative and non-oxidative pathways. The oxidative pathway involves alcohol dehydrogenase (ADH), microsomal cytochrome P450 enzymes (CYP450), and peroxidases.

### 2.2 Metabolic dysfunction-associated fatty liver disease

Currently, metabolic dysfunction-associated fatty liver disease (MAFLD) affects one-quarter of the global adult population (22, 23). MAFLD can also occur in individuals without obesity or metabolic syndrome, possibly due to certain metabolic disorders, such as insulin resistance(IR) or increased cardiovascular risk. Excess fatty acids lead to IR and liver steatosis, which eventually cause liver cell damage, inflammation, fibrosis, and other pathological changes due to oxidative stress and lipid peroxidation (24). The mechanisms of MAFLD pathology are varied, including oxidative stress, ER stress, and lipotoxicity (25).

#### 2.2.1 Lipid accumulation

When energy intake exceeds consumption, the excess energy is stored as lipids in organs throughout the body (26–28). MAFLD is formed through ectopic lipid accumulation. The steatosis in MAFLD is triggered by the excessive synthesis of triglycerides (TG) in liver cells, with 60% of the substrates for synthesis coming from white adipose tissue (WAT), 26% from *de novo* lipogenesis (DNL), and 15% from high-fat and high-sugar diets (29, 30). Insulin has an anti-lipolytic effect, mediating the storage of TG in adipose tissue and promoting the esterification and storage of fatty acids (31). Therefore, IR is a key factor in MAFLD. In the IR state, insulin’s anti-lipolytic function weakens, WAT is broken down, leading to a large release of free fatty acids (FFA) (32). Excess FFAs are then stored in the liver as TG, forming ectopic lipid deposits and leading to MAFLD (33).

**Figure 2:**
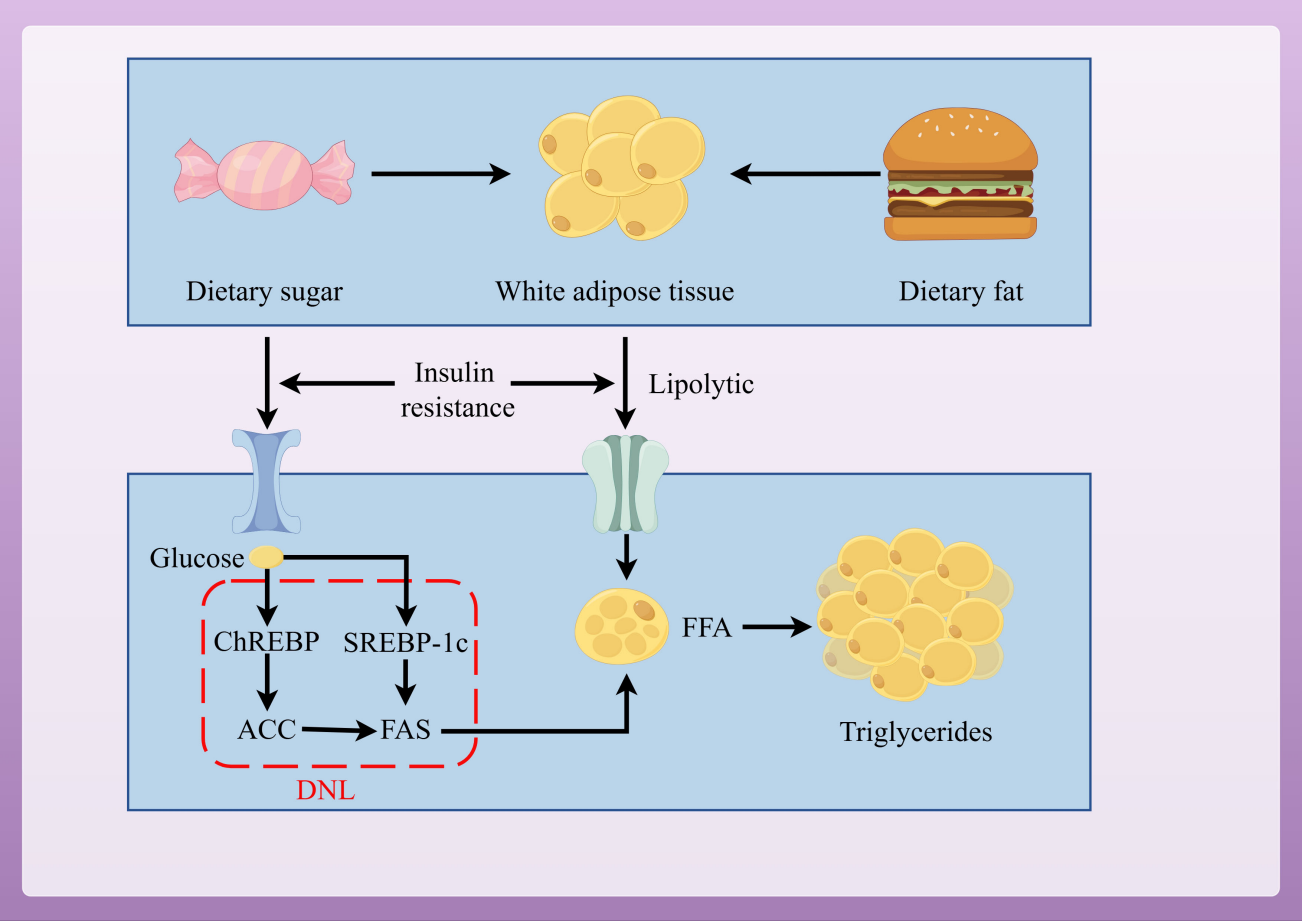
Pathways of lipid accumulation leading to MAFLD.

DNL is a key pathway for promoting lipid accumulation and is closely related to IR (34). DNL is regulated by sterol regulatory element-binding protein-1c (SREBP-1c) and carbohydrate response element-binding protein (ChREBP) (35, 36). IR activates SREBP-1c to promote DNL in liver cells (37, 38). Increased glucose concentrations activate ChREBP to regulate the expression of acetyl-CoA carboxylase (ACC) and fatty acid synthase (FAS), thus promoting DNL in liver cells (**Figure 2**) (39).

Dietary factors play a crucial role in the development of MAFLD (40, 41) A diet high in fats and sugars increases the expression of genes related to liver fibrosis, inflammation, ER stress, and adipocyte apoptosis (42). Animal models and human studies have shown that fructose has selective liver metabolism and triggers liver stress responses, including activating c-Jun N-terminal kinase (JNK) and IR, promoting liver fat accumulation, impairing fatty acid oxidation (FAO), and leading to liver inflammation and fibrosis (43, 44).

#### 2.2.2 Oxidative stress

DNL converts excess carbohydrates into fatty acids, which are then esterified into triglycerides (TG) and stored in liver cells. When energy is insufficient, TG is used to supply energy through β-oxidation (45). However, an increase in FFA in the liver impairs β-oxidation and mitochondrial function, leading to oxidative stress (46).

Peroxisomes are the first enzymes in the fatty acid β-oxidation system. Peroxisome proliferator-activated receptor alpha (PPARα) regulates the activity of three interconnected liver fatty acid oxidation systems: mitochondrial and peroxisomal β-oxidation and microsomal ω-oxidation pathways (47). Continuous activation of PPARα can alleviate MAFLD by enhancing FAO and reducing ROS levels (48, 49). However, many studies have found that excessive activation of PPARα leads to overconsumption of liver energy, disproportionately increasing H2O2 and triggering an inflammatory response (50).

**Figure 3:**
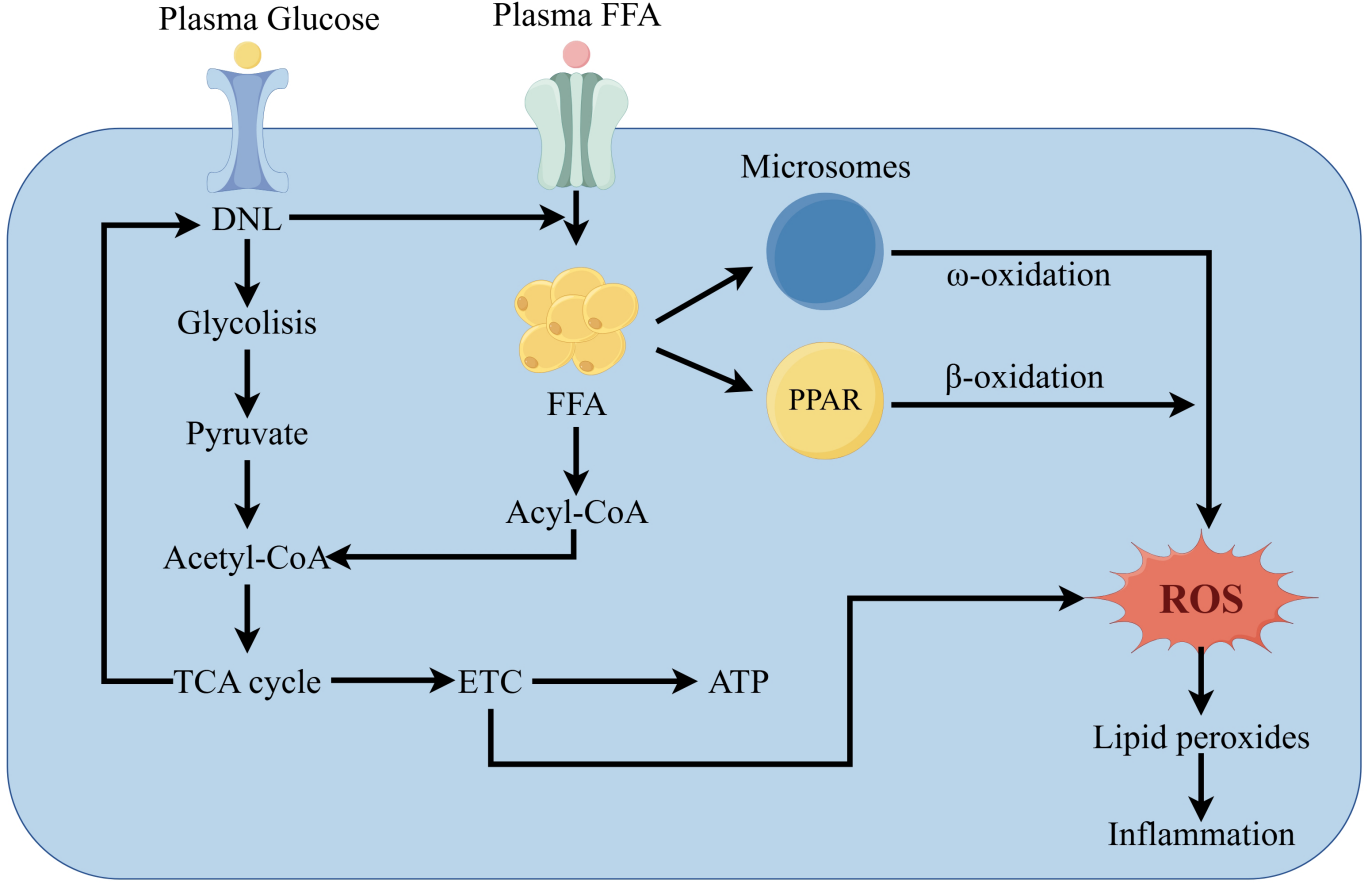
Pathways of MAFLD development due to oxidative stress.

MAFLD patients show damage to mitochondrial ultrastructure, reduced respiratory chain complex activity, and impaired ATP synthesis (51). Mitochondria play a crucial role in FAO and energy supply while producing large amounts of ROS (52). Mitochondrial dysfunction results from damage to the electron transport chain (ETC). Over-reduction of components of the mitochondrial respiratory chain leads to abnormal reactions between electrons and oxygen, increasing ROS (53). Moreover, ROS oxidize lipid deposits, releasing lipid peroxides that damage liver cells. In liver cells, ROS and lipid peroxides further disrupt the respiratory chain, directly or indirectly causing oxidative damage to the mitochondrial genome, which leads to more ROS production, creating a vicious cycle, ultimately leading to inflammation (**Figure 3**) (54).

#### 2.2.3 Endoplasmic reticulum stress

ER stress represents a protective cellular reaction, triggering the unfolded protein response (UPR) in an effort to restore protein homeostasis. As lipid accumulation increases, ER stress results in a buildup of unfolded proteins, triggering the UPR (55) The UPR is mediated by protein kinase RNA-like ER kinase (PERK), inositol-requiring enzyme 1 (IRE1), and activating transcription factor 6 (ATF6) (56), all of which regulate lipid storage in the liver (57). PERK-mediated phosphorylation of eukaryotic initiation factor 2α (eIF2α) transiently reduces translation, while activating transcription factor 4 (ATF4) induces the expression of the gene CCAAT/enhancer-binding protein homologous protein (CHOP) (58). ATF6 and IRE1 promote the expression of X-box binding protein-1 (XBP1) and mediate inflammation through the JNK signaling pathway (59, 60). Additionally, IRE1 can directly activate JNK, which in turn activates TNF receptor-associated factor 2 (TRAF2), promoting apoptosis (61).

#### 2.2.4 Lipotoxicity

Lipotoxicity refers to the toxic effects caused by the excessive deposition of lipids and their metabolites in non-adipose tissues (62). When the concentration of lipotoxic substances in liver cells exceeds the transport capacity of the liver, the damage to liver cells worsens, and The more that the disease advances, the more stages it will progress to. One of the reasons that IR will stem from the disease in MAFLD is due to a surge of FFA in plasma. Thus, the liver cells undergo an overload of FAO, which results in a great production of ROS, damage to mitochondria, ER stress, and the addition of inflammation. The lipotoxicity caused by lipid accumulation drives the further progression of the disease.

**Figure 4:**
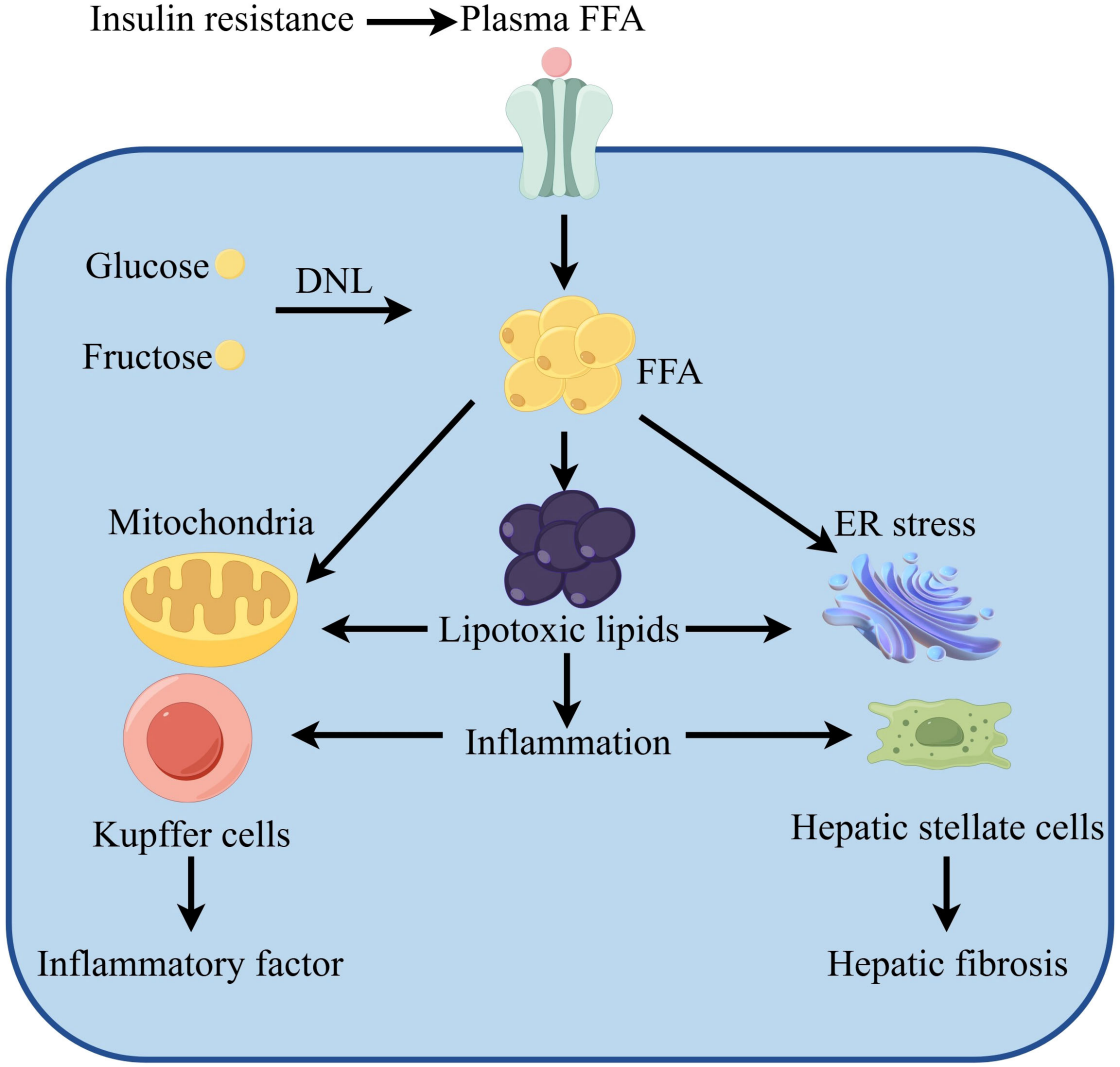
Lipotoxicity causes MAFLD.

Liver parenchymal cells are mainly composed of hepatocytes. Hepatic stellate cells (HSCs) and Kupffer cells (KCs) are examples of non-parenchymal cells that also have a vital job in the development of NASH. Liver fibrosis is a major cause for the progression of NASH and usually stems from HSCs. It has been found that the activation of toll-like receptor 4 (TLR4) by lipotoxic substances promotes inflammation and fibrotic signaling in HSCs (63). KCs regulate inflammatory responses in the liver microenvironment and contribute to liver disease progression by secreting pro-inflammatory cytokines. In patients with NASH, elevated levels of oxidized LDL trigger inflammation in KCs (**Figure 4**) (64).

### 2.3 Viral hepatitis

Viral hepatitis generally includes five types: hepatitis A, B, C, D, and E. When liver enzymes increase in the serum, the liver may be under viral attack. Cytotoxic T cells play a key role in virus clearance during the acute phase of hepatitis (65).

During viral infection, the virus activates cytotoxic T lymphocytes (CD8T cells), which produce virus-specific CD8T cells. Activated virus-specific CD8T cells differentiate into effector cytotoxic T lymphocytes, which specifically kill virus-infected cells, leading to liver damage and possibly progressing to liver cancer (66, 67).

Viral hepatitis includes both acute and chronic forms. In chronic infections, the body’s immune response is impaired as the target virus persists. If the host immune response fails to clear the virus, it leads to immune evasion due to genetic mutations, and viral proteins suppress immune responses. Acute hepatitis can typically resolve on its own if the liver cell damage is within recoverable limits. However, severe acute hepatitis may require medication to prevent progression to chronic hepatitis.

### 2.4 Cirrhosis

Chronic liver diseases progress to cirrhosis through an intermediate stage of liver fibrosis. Liver fibrosis occurs when ECM proteins (primarily cross-linked types I and III collagen) accumulate, replacing damaged normal tissue with fibrotic scars (68). Key mechanisms of liver fibrosis include chronic hepatocyte damage, epithelial or endothelial barrier injury, the release of inflammatory cytokines, recruitment of bone marrow-derived inflammatory cells, macrophage production of TGF-β, and activation of hepatic myofibroblasts that secrete type I collagen (COL1A1) to produce excessive ECM (69).

(a) HSCs reside in the liver’s space of Disse and exist in a quiescent phenotype. Quiescent HSCs are the main storage site for vitamin A (70, 71), and maintaining this quiescent phenotype is critical (72). Upon liver injury, quiescent HSCs downregulate the expression of vitamin A, GFAP, and PPARγ, becoming activated (73).

Upon stimulation by fibrogenic mediators, HSCs upregulate α-smooth muscle actin and other intracellular microfilaments of myofibroblasts. Activated HSCs migrate to the injury site and secrete ECM, forming fibrotic scars (74).

TGF-β is the most potent fibrogenic cytokine (75). COL1A1, COL1A2 proteins, Activin, and Pai1 genes are targets of TGF-β. Additionally, IL-6 and IL-17 can induce COL1A1 transcription through the JAK-STAT3 signaling pathway (76). Connective tissue growth factor (CTGF) and IL-13 promote COL1A1 expression in activated HSCs via the TGF-β1 pathway81. Research shows that inflammation is an essential factor for fibrosis; signals from damaged hepatocytes alone are insufficient to directly activate HSCs and cause fibrosis.

(b) Inflammation plays a crucial role in the pathogenesis of liver fibrosis. Neutrophils are often recruited to the injured liver as the first responders to clear apoptotic liver cells (77). Neutrophils release free DNA, which has a strong pro-inflammatory effect (78). Increased levels of neutrophil chemokines (IL-8, IL-18, IL-17, CCL3, CCL4, and CXCL2) accelerate liver fibrosis progression in mice (79, 80).

The activation of macrophages capable of producing TGF-β is a key factor in liver fibrosis. Kupffer cells are the main source of TGF-β, and they possess phagocytic and anti-inflammatory functions (81). Overexpression of myeloid TGF-β genes spontaneously induces fibrosis in target tissues and organs (including the liver), indicating that TGF-β is a crucial mediator of fibrosis. Furthermore, the deletion of IL-6, TNF, or IL-1β genes reduces liver fibrosis progression, as these cytokines synergize with TGF-β (82, 83).

(c)Viral hepatitis (especially hepatitis B and C), alcoholic liver disease, and MAFLD can all lead to liver fibrosis and eventually cirrhosis. For example, ALD is a leading cause of cirrhosis and liver failure. ALD progresses from fatty degeneration to steatohepatitis, fibrosis, and HCC (84). Alcohol induces liver injury both directly (through toxic ethanol metabolites) and indirectly (via cytochrome P4502E1, an alcohol-metabolizing enzyme) (85). Alcohol activates SREBP1 or SREBP2-dependent fatty acid and cholesterol synthesis (84), leading to the accumulation of lipid droplets in the liver, the formation of Mallory bodies in ballooned hepatocytes, and alcohol-induced liver injury (86). Alcohol-induced hepatotoxic damage is associated with the upregulation of IL-8, IL-17, CXCL1, neutrophil infiltration, recruitment of bone marrow-derived or liver macrophages to the alcohol-injured liver, TGF-β secretion, and activation of myofibroblasts, which further produce excessive ECM proteins that contribute to liver fibrosis (86–88).

## 3 Hepatocellular carcinoma

HCC is a cancer that originates in the cells of the liver. Its pathogenesis involves multiple molecular defects, including cell cycle dysregulation, changes in DNA methylation, chromosomal instability, immune modulation, epithelial-to-mesenchymal transition, increases in HCC stem cells, and dysregulation of miRNAs (89). While the specific mechanisms driving HCC differ based on the underlying etiology, the usual progression involves liver injury, followed by chronic inflammation, fibrosis, cirrhosis, and ultimately HCC. The release of molecular mediators, including damage-associated molecular patterns (DAMPs) and pathogen-associated molecular patterns (PAMPs), by viral particles activates pattern recognition receptors (PRRs) present on immune cells. These PRRs encompass Toll-like receptors (TLRs), C-type lectin receptors, NOD-like receptors, and retinoic acid-inducible gene I (RIG-I)-like receptors, thereby triggering inflammation. This chronic inflammation can lead to fibrosis and eventually cirrhosis (90). Research into molecular mechanisms related to the development of liver cancer has the potential to identify therapeutic targets.

### 3.1 IGF pathway

Aberrant IGF signaling is critically involved in the pathogenesis and carcinogenic processes of HCC, especially in insulin resistance-related HCC. Insulin and hyperinsulinemia promote the synthesis and bioactivity of IGF-1 and IGF-2, regulating energy-dependent growth processes (91).

IGF-1 has a higher affinity for IGF-1 R, which is associated with the development of precancerous lesions (92). The binding of IGF-1 to IGF-1 R can regulate stem cell pluripotency and differentiation, triggering cell proliferation, organ development, and tissue regeneration (93). Additionally, imbalances in IGF-1/IGF-1 R signaling can activate MAPK and c-JNK pathways, promoting HCC cell proliferation and inhibiting apoptosis. IGF-1 also promotes angiogenesis by increasing the production of VEGF (94). Plasma levels of IGF-2 are elevated in patients with obesity, cirrhosis, and HCC (95). During hepatocarcinogenesis, IGF-2 exerts various carcinogenic functions by binding to IGF-1 R, such as inhibiting apoptosis, promoting HCC cell proliferation and migration, and activating angiogenesis (96). Studies indicate that IRS-1 is an oncogene with higher expression levels in HCC (97). Hyperinsulinemia and increased IGF receptor activation lead to the phosphorylation of IRS-1, triggering the activation of multiple cytokine pathways, including the PI3 K/AKT/mTOR and MAPK cascades, which regulate the cell cycle and may potentially enhance tumor progression in HCC (98).

### 3.2 Wnt/β-catenin pathway

Wnt/β-catenin signaling pathway is one of the most important pathways for cell fate differentiation and the overall maintenance of liver metabolism and homeostasis (99). In patients with cirrhosis and HCC, Wnt activity is frequently overactivated. Abnormal activation of Wnt/β-catenin signaling is a hallmark of various liver pathologies, playing a role in nearly every aspect of liver biology (100).

At the heart of the Wnt signaling cascade lies β-catenin, a protein produced from the *CTNNB1* gene. The pathway centers on the interaction between Wnt ligands and the Frizzled/LRP co-receptor complex, leading to abnormal accumulation of β-catenin in the nucleus and the expression of multiple transcriptional targets, including genes responsible for proliferation (e.g., MYC), anti-apoptosis (e.g., BIRC5), epithelial-mesenchymal transition (e.g., Snail), invasion (e.g., MMP), angiogenesis (e.g., VEGF), and inflammation (e.g., IL-6) (101). β-catenin also functions in cell-cell adhesion as a component of adherens junctions (100). Hepatic stellate cells (HSCs) express several Wnt receptors, with components like Wnt 3a and Wnt 5a promoting HSC activation, which is crucial in fibrosis development and progression (102). Therefore, activation of the Wnt/β-catenin pathway regulates tissue development and regeneration, as well as HCC tumorigenicity and metastatic potential (99). Increasing evidence links Wnt/β-catenin to human inflammation (e.g., HBV and HCV) and metabolic dysfunction (103). It can regulate liver function by modulating Supplementary regulating cytokines like FAS, and the PPAR family (104). Additionally, Wnt/β-catenin pathway plays an essential role in HCC by mediating communication between the distinct components of the TME, such as immune cells, stem cells, and non-cellular constituents (105).

### 3.3 JAK/STAT pathway

As a key downstream signal transducer for numerous cytokines (such as IL-6) and growth factors (such as EGF), the JAK/STAT pathway exhibits dysregulation in inflammatory conditions and HCC.JAK and STAT regulate cell development, with persistent activation of STAT leading to harmful pathological effects (106, 107).

Disruption of the GH/JAK2/STAT5 signaling pathway, a result of inhibited growth hormone (GH) secretion (brought on by obesity, inflammation, and excessive glucose), results in increased lipid accumulation in the liver, further leading to MAFLD and subsequently HCC (108). STAT3 is strongly linked to liver injury, playing a significant role in the genesis of liver diseases; a common activator of STAT3 is IL-6. Activating the IL-6/JAK/STAT3 signaling cascade within the liver amplifies inflammation and immune responses, furthering the development of HCC (109).

### 3.4 PI3K/AKT pathway

Upon receptor binding by insulin and inflammation, the PI3K/AKT pathway is activated, acting as an essential oncogenic mechanism controlling metabolism, cell growth, and survival. Inflammation is worsened by dysregulated PI3K/AKT signaling, which can then lead to type 2 diabetes mellitus and development of HCC. AKT maintains hepatic lipid homeostasis through regulation of lipid metabolism. The PI3K/AKT signaling process triggers the creation of genes for proteins and transcription factors that play a role in DNL, acetyl-CoA carboxylase α (ACCα) and SREBP1 for example (110, 111).

### 3.5 MAPK pathway

MAPK pathway comprises a family of mitogen-activated protein kinases, including stress-responsive MAPK, c-JNK, and p38 MAPK (112).

High activation of JNK is evident in HCC, which is related to the severity of liver histological activity and facilitates carcinogenesis (113). Increases of ROS, FFA, and TNF-α during chronic inflammation and obesity triggers activation of JNK in hepatocytes and macrophages, thus increasing production of inflammatory cytokines that drives inflammation, apoptosis, liver injury and fibrosis, and hepatic IR, thereby highlighting the metabolic effects of the JNK pathway (114). Macrophage overactivation of JNK is important for pro-inflammatory differentiation and tissue infiltration, while JNK1 deficiency within macrophages prevents hepatic IR. JNK directly contributes to reducing fatty acid oxidation and increasing the potential of steatosis by inhibiting hepatic PPARα and other genes that it targets. Activation of apoptotic proteins, such as Bcl-2-L-11, BAD, and Bcl-2-L-4, result in the initiation of lipotoxicity and apoptosis through the function of JNK (115).

p38α/β MAPK stimulates generation of hepatocytes by activating pro-apoptotic genes, such as PEPCK, G6Pase, and PGC-1α. Activation of p38α MAPK has been recently shown to drive ER stress and IR, accelerating the development of NASH (112); MAFLD patients who are obese display increased levels of p38α MAPK, which leads to HCC (116).

### 3.6 AMPK pathway

An intracellular energy sensor is the AMPK pathway, also known as AMP-activated protein kinase, which plays an essential role in maintaining energy homeostasis while also taking part in various biological processes. Activation of AMPK increases if there is nutrient deprivation; however, it decreases if there is chronic inflammation and MAFLD (117). In order to combat liver injury and fibrosis, increasing AMPK activity is a viable therapeutic plan. The loss of AMPK activity would exacerbate liver injury and fibrosis. The prevention of HSC activation, proliferation, and migration can improve liver fibrosis by activating AMPK, in addition to reducing fibrotic stimuli and inhibiting the expression of fibrotic genes (118). Cell proliferation is regulated through AMPK’s inhibition of mTOR signaling (119).

### 3.7 NF-κB and Toll-like pathway

Key inflammatory pathways involved in HCC are the NF-κB and Toll-like receptor (TLR) pathways (120, 121). Chronic inflammation, which is a product of saturated fatty acids, activates pro-inflammatory pathways in adipocytes and macrophages using a mechanism dependent on TLR4 (115). After TLR signaling, transcription factors like NF-κB and AP-1 are activated, increasing secretion of inflammatory cytokines like IL-6, IL-1β, and TNF-α. This increase in pro-inflammatory cytokines that occur in hepatocytes will lead to insulin resistance, liver cell damage, and also the progression of MAFLD, NASH, and HCC. The gut microbiota’s dependence on TLRs is an important characteristic when looking at the relationship between inflammation and obesity. Also, mice without TLR5 have a distinct gut microbiota profile, showing susceptibility to metabolic syndrome (122).

NF-κB is a transcription factor that is crucial in the processes of inflammation, immunity, cell proliferation, and how liver injury, fibrosis, and HCC occurs (123). IKKα/IKKβ, a complex that directly activates NF-κB, is associated with downstream gene expression of TLRs and cytokines. Broadly, NF-κB has many responsibilities in various cellular compartments; it has been seen to affect hepatocyte survival, inflammation that occurs in KCs, and also the survival, inflammation, and activation of HSCs (124). If NF-κB regulates HSC survival, it will also promote the induction and secretion of inflammatory chemokines such as CCL2 and CCL3. However, NF-κB has a protective effect in the liver. Significant inhibition of NF-κB has been shown to cause hepatocyte apoptosis (125).

### 3.8 p53 pathway

Integrating cellular stress responses, metabolism, and cell cycle regulation, the tumor suppressor gene p53 is a key regulatory element in both liver homeostasis and dysfunction (103, 126). Moderate and transient p53 activation inhibits both liver lipid accumulation and inflammation under normal conditions. However, during cellular stress stemming from inflammation or NASH, excessive p53 activation can trigger IR, lipid accumulation, inflammation, and oxidative stress through various mechanisms, thereby increasing the risk of HCC (127, 128).

Elevated p53 levels can worsen the release of pro-inflammatory cytokines, which in turn contributes to metabolic abnormalities facilitating HCC initiation and progression (129, 130). For instance, systemic IR is triggered by p53 activation during hyperlipidemia or excessive caloric intake. The formation of white and brown adipose tissue is critically regulated by p53, functioning as a suppressor of adipocyte pre-differentiation (131). Within AT, NF-κB signaling leads to the expression of pro-inflammatory adipokines following p53 activation, resulting in hepatic steatosis, IR, and inflammation. Conversely, reducing p53 activity can diminish inflammation and ease hepatic steatosis (132). Further, as a major positive regulator of lipid metabolism in hepatocytes, p53 plays a role in lipotoxicity-mediated NASH progression (133). Increased hepatocyte apoptosis, driven by p53 activation, contributes to liver fibrosis, and eliminating p53 completely negates this fibrotic phenotype, indicating significant implications for HCC progression (134).

## 4 Current status and prospects of liver cancer treatment

Ranking at fifth most common is HCC, hepatocellular carcinoma, when looking at cancers worldwide. It is the cause of death for the third leading cause of cancer-related deaths (135). Currently, tumor resection is the most effective form of treatment for this cancer; however, in postoperative recovery, the tumor can have high recurrence (136). In addition, there are only two clinical drugs that specifically target HCC. Those drugs, sorafenib and lenvatinib, can extend overall survival by approximately 2-3 months (135). Therefore, new treatments for HCC are urgently needed, and macrophage-targeted therapy and immunotherapy for liver cancer have become research hotspots.

### 4.1 Liver cancer immunotherapy

Cancer immunotherapy triggers systemic and lasting anti-tumor responses, making it a promising option for treating HCC. Immune checkpoint inhibitors (ICIs) targeting cytotoxic T-lymphocyte antigen-4 (CTLA-4), programmed cell death protein-1 (PD-1), or its ligand programmed cell death-ligand 1 (PD-L1) have demonstrated therapeutic benefit in HCC 144. Beyond ICIs, adoptive cell therapy, chimeric antigen receptor (CAR)-modified immune cells, engineered cytokines, and therapeutic cancer vaccines represent increasingly viable immunotherapy approaches in clinical settings (136, 137). The challenges and future directions in this research field are discussed below.

Immune Checkpoint Inhibitors (ICIs)

Expressed on immune cells, a collection of molecules known as immune checkpoints finely regulate the level of immune activation. An important function of these checkpoints is to prevent autoimmune reactions, a condition characterized by the immune system attacking the body’s own healthy cells (138). Tumor cells can over-activate immune checkpoints, leading to immune system dysfunction. ICIs can relieve this inhibition, reactivating immune cells to attack and destroy cancer cells.

ICIs are monoclonal antibodies designed to disrupt the interaction between immune checkpoint proteins and their respective ligands. By blocking T-cell inactivation and reinvigorating immune recognition and attack, ICIs amplify the anti-tumor immune response. Common ICI targets currently include PD-1, PD-L1, and CTLA-4 (139). PD-1 is found on the surface of most immune cell types, predominantly on activated T cells, NK cells, regulatory T cells (Tregs), myeloid-derived suppressor cells (MDSCs), monocytes, and dendritic cells (DCs). This protein can bind to its ligands PD-L1 and PD-L2, which are expressed in many tumors, including HCC, transmitting inhibitory signals to T cells and inducing immune evasion by tumor cells (140).

**Figure 5:**
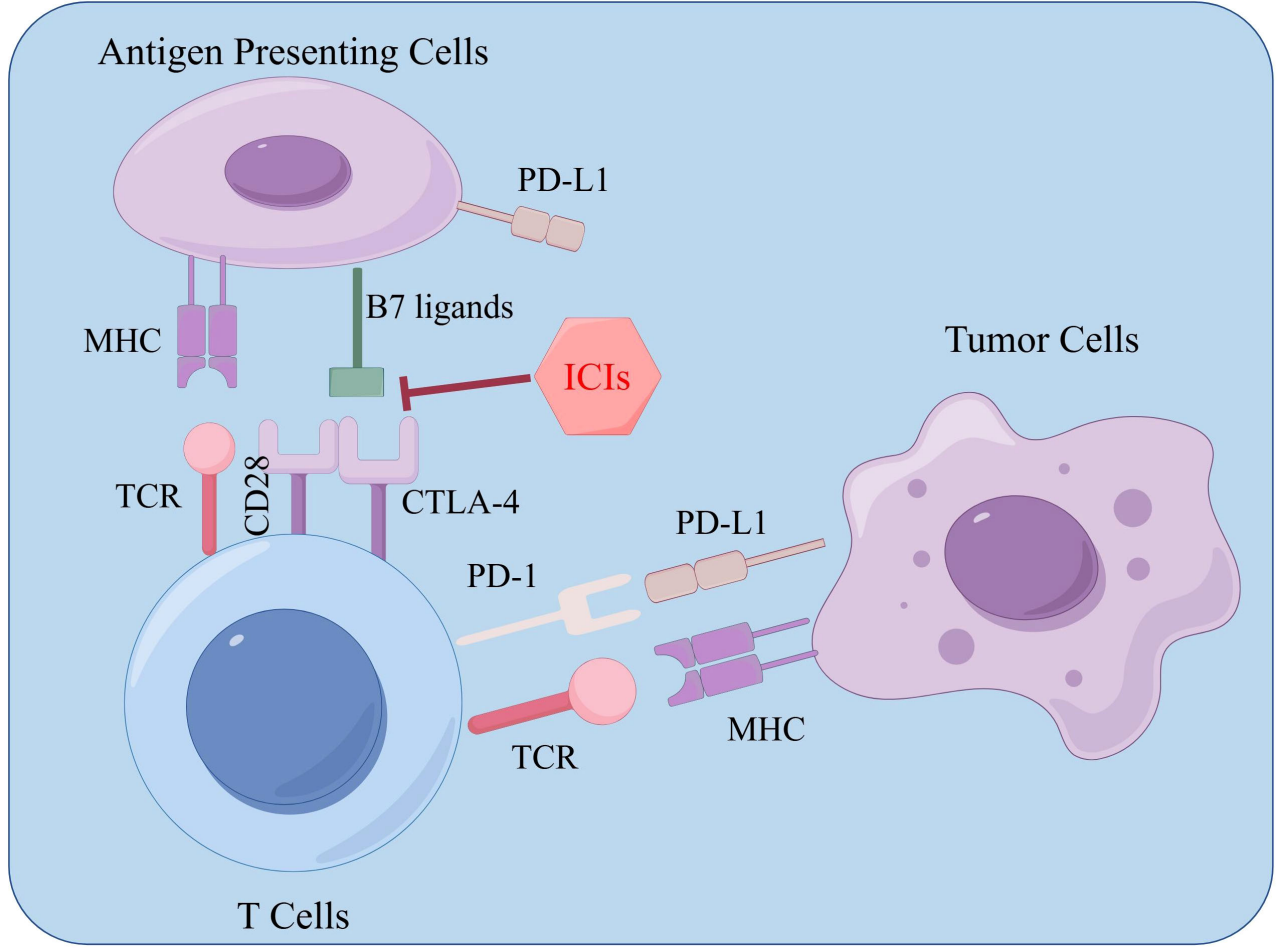
Immune checkpoint inhibitor-related target effects.

Acting as a transmembrane receptor on T cells, CTLA-4 is expressed mainly on dendritic cells and activated T cells. It participates in the negative regulation of the immune response after the B7 molecule binds to it. The B7 molecule ligand can be bound by both CTLA-4 and CD28 (141). Compared to CD28, the affinity for the ligand is 20-100 times higher in CTLA-4. If the ligand binds to it, then it inhibits cell proliferation, stops the production of cytokines, and prevents cell cycle progression. Competitively, CTLA-4 blocks CD28 for the B7-1/B7-2 ligand; because of this, CD28 co-stimulation is unable to work.(**Figure 5**).

### 4.2 Anti-tumor cell therapy

ACT uses immune cells from the patient or healthy donors to combat cancer and has become a viable option for cancer treatment (142). Compared to targeted drugs, ACT can be activated and replicated within the body, producing a lasting anti-tumor effect (143).

Traditional immune cell therapies, such as CIK (cytokine-induced killer) cells, involve culturing a patient’s immune cells outside the body and reinfusing them to target and kill tumor cells. The key components of CIK cells are NKT cells, natural killer NK cells, and cytotoxic T lymphocytes (CTLs). Leveraging adhesion molecules, CIK cells recognize tumor cells and induce lysis independent of major histocompatibility complex (MHC) restriction (144). However, traditional immune cell therapy lacks specificity, limiting its effectiveness. The key to enhancing immune cell-mediated tumor killing lies in improving the immune cells’ ability to recognize tumors.

**Figure 6:**
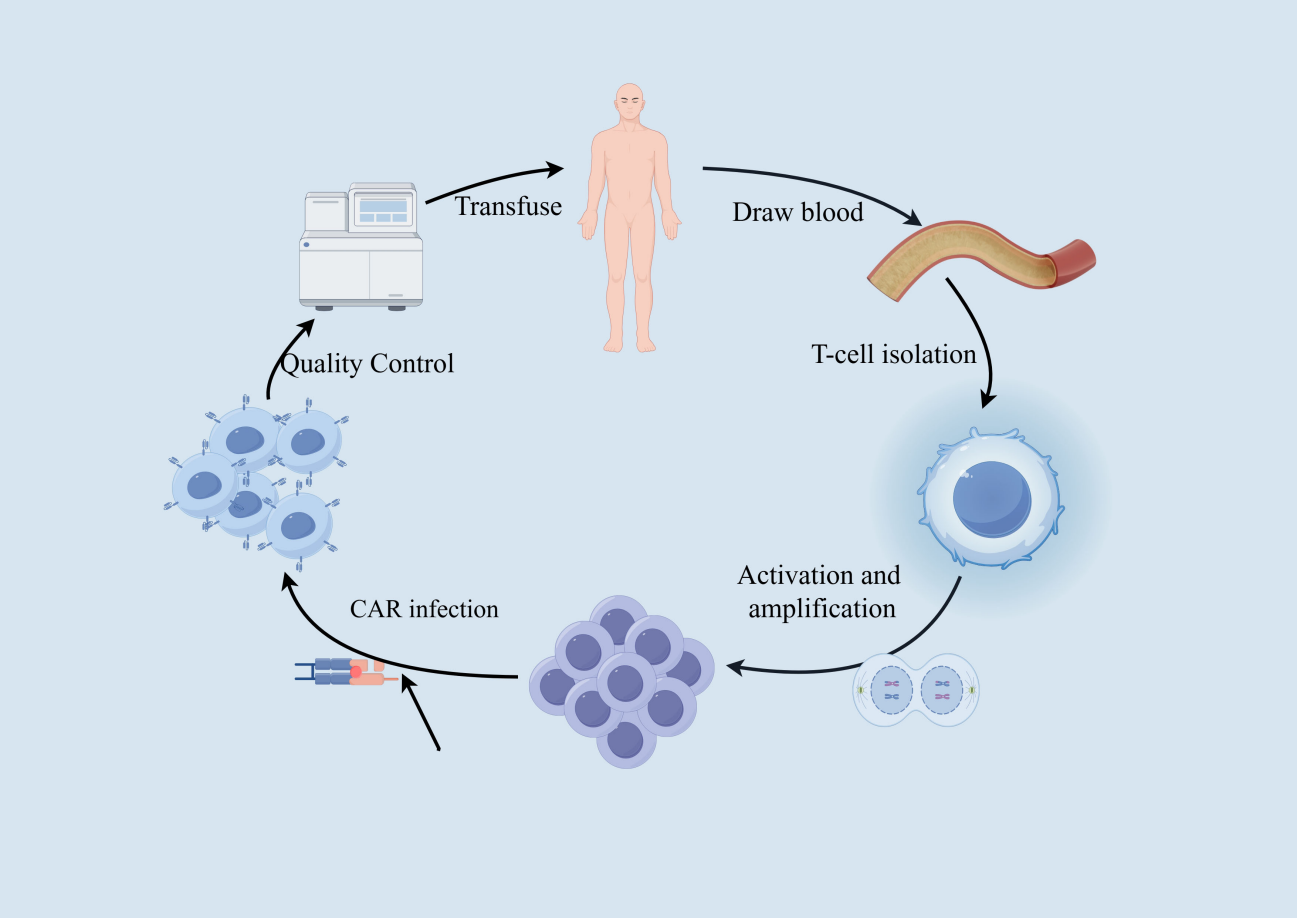
CAR-T treatment process.

Most tumor cells express certain tumor-specific or tumor-associated antigens (TSA or TAA). By combining the “antigen recognition domain (scFv)” of antibodies that recognize these tumor antigens with components that promote T-cell proliferation, and transducing them into the patient’s T cells using gene transfer, the T cells are made to express a CAR. Once the patient’s T cells are “reprogrammed,” they can produce large quantities of tumor-specific CAR-T cells upon contact with target cells, achieving specific killing of tumor cells. This is known as CAR-T cell therapy. By introducing a synthetically designed CAR molecule into T cells, CAR-T cells gain a new targeted activation function, and once reinfused into the patient, these CAR-T cells are no longer MHC-restricted. Instead, they are activated by binding to target antigens, efficiently killing tumor cells. This process is illustrated in **Figure 6**.

While still under development (145), due to the complexity of solid tumors and their heterogeneity, finding specific targets for CAR-T therapy for liver cancer continues to be the focus of research. One research avenue looks towards using GPC3 (glypican-3) as a target in order to kill HCC (146). Using Tet-On inducible CD147-CAR-T cells has also shown promise in that these cells have successfully destroyed several HCC cell lines and inhibited the growth of cancer in xenograft models (147). However, all of these targets can be also found in other parts of the body leading to toxicity. The research moving forward lies in finding more specific antigens, as well as improving the efficacy and safety of CAR-T therapy.

Building on CAR-T therapy, research has also extended to CAR-NK immunotherapy (148, 149), TCR-T (150, 151), and other advanced approaches that continue to be explored and developed.

### 4.3 Macrophage-targeted therapy in liver cancer

Depending on the signals present in their microenvironment, macrophages can adopt different polarized states. Primarily, based on their activation state and function, they are grouped as classically activated, pro-inflammatory M1 macrophages, or selectively activated, anti-inflammatory M2 macrophages (152). The polarization of macrophages towards an M1 phenotype can be triggered by IFN-γ, LPS, or GM-CSF. These M1 macrophages, by secreting inflammatory cytokines like IL-1, contribute to inflammatory responses, defend against intracellular pathogens, and exhibit anti-tumor effects (153, 154).

In contrast, M2 macrophages are induced by IL-4, IL-13, and exhibit high expression of CD206, enhanced phagocytic capacity, and secrete anti-inflammatory cytokines like IL-10 and TGF-β, facilitating Th2 cell differentiation, immune regulation, repair functions, wound healing, angiogenesis, and promoting tumor progression (155).

**Figure 7:**
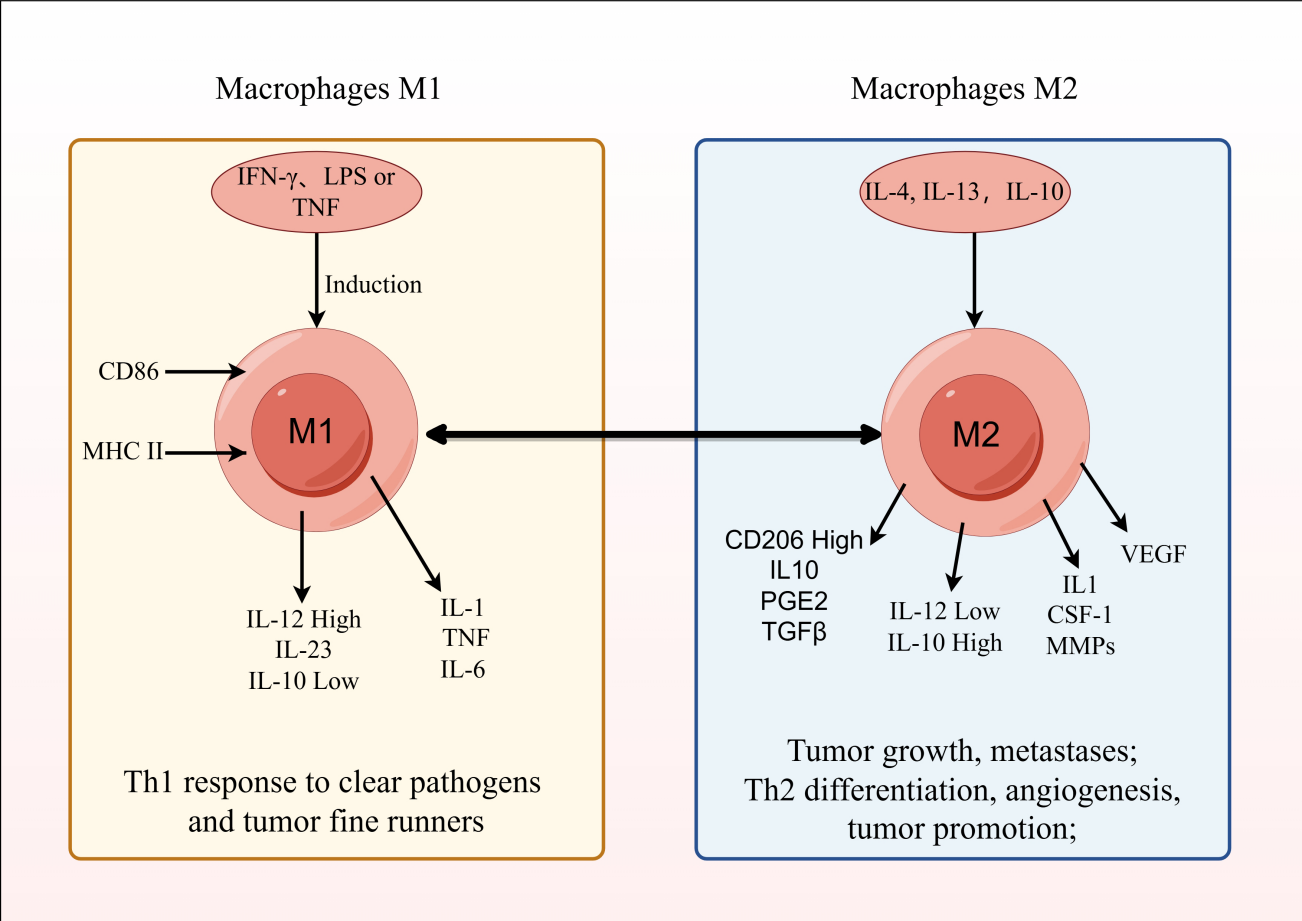
Mechanism of action of tumor-associated macrophages.

Among them, M2 TAMs contribute to enhancing the stem cell-like properties of cancer cells in liver cancer (156, 157). They participate in the growth of tumor microvessels and lymphatic vessels by secreting VEGF (vascular endothelial growth factor) and EGF, promoting tumor cell proliferation (158). Additionally, they secrete IL-1, CSF-1, MMPs, etc., which facilitate tumor cell metastasis and invasion (159, 160). M2 TAMs are also involved in tumor immune evasion regulation by producing IL-10, PGE2, TGF-β, and they can promote tumor growth by regulating tumor cell metabolism (**Figure 7**) (161, 162).

As an important cell type that promotes tumor growth and metastasis, M2 TAMs can serve as crucial therapeutic targets. Drug development targeting tumor-associated macrophages can be approached in three ways: inhibiting the production of tumor-associated macrophages and promoting their exhaustion (163, 164); suppressing the recruitment of TAMs; and reprogramming TAMs to shift from a tumor-suppressive immune state to a tumor-promoting immune state (transforming M2 to M1) (165, 166).

Besides CAR-T cell therapy, clinical trials are exploring CAR-M therapy. Genetically engineered macrophages can target CD19 and CD22 antigens to find tumor cells (167). These CAR-M cells then eat the tumor cells, release chemicals to change the tumor’s environment, present tumor antigens to T cells, and boost immune responses (168). Studies in solid tumors show macrophages effectively destroy tumor cells via SYK (spleen tyrosine kinase) (169). A viral vector, Ad5/f35, can make macrophages stay in an M1 state in mice, improving T cell activity and stopping solid tumor growth. However, finding specific targets on liver cancer cells and engineering macrophages within the liver environment is hard due to the diversity of liver cancers.

Attention must also be paid to the potential off-target toxicity and immunogenicity of these treatments (170–172). As CAR-M technology continues to evolve, developing safer, more reliable, and more effective CAR-M approaches is essential for translating it into clinical practice. Whether combining CAR-M with CAR-T, multi-target kinase inhibitors, and ICIs can synergistically enhance tumor suppression remains an area for future research.

### 4.4 Traditional Chinese medicine in liver cancer treatment

In Traditional Chinese Medicine (TCM), liver cancer falls under concepts such as “jaundice” and “accumulation.” In the early stages, liver cancer is often characterized by excess conditions, gradually progressing to a combination of excess and deficiency, and ultimately becoming deficiency with excess signs in advanced stages, closely related to pathological factors such as blood stasis and damp-heat toxins (173). The research value of TCM in treating liver cancer is of significant importance in modern pharmacology. For instance, ginsenoside Rb1 can induce apoptosis and inhibit tumor progression by mediating mitochondrial autophagy (174). Ginseng polysaccharides can induce apoptosis in liver cancer cells via the ERK pathway, potentially blocking tumor cells at the G0/G1 phase and inhibiting their proliferation (175). Additionally, interventions with different concentrations of Astragalus polysaccharides on SMMC-7721 cells may reduce tumor cell migration and invasion by inhibiting the activation of the JAK/STAT signaling pathway (176). Studies have also shown that polysaccharides from Half-leaf Mimosa can inhibit tumor growth in H22 tumor-bearing mice potentially downregulating the expression of VEGFA and further suppressing the VEGF signaling pathway to inhibit tumor angiogenesis (177),. Many other traditional Chinese medicines have significant research value in liver cancer treatment, particularly the combination of TCM formulas, which may yield synergistic effects in therapy.

With the increasing incidence of HCC, the hepatoprotective effects of TCM are becoming increasingly important. Compared to conventional drugs, TCM offers advantages such as wide availability, lower cost, greater stability, and fewer side effects. Furthermore, numerous studies have demonstrated the liver-protective effects of TCM extracts through anti-lipid peroxidation mechanisms. Both hesperidin and Bicyclol have shown promise in addressing hepatic steatosis. Hesperidin, in *in vitro* and *in vivo* settings, has been shown to alleviate steatosis by upregulating antioxidant levels through PI3K/AKT-Nrf2 and inhibiting NF-κB-mediated inflammation (178). Bicyclol, found as an extract from *Schisandra chinensis*, possesses a wide variety of pharmacological activities. Notably, Bicyclol lessens tetracycline-induced steatosis while also ameliorating hepatic lipid accumulation and physalin-induced steatosis (179).

Therapies derived from TCM present certain obstacles for treatment of HCC, yet the prevention and early management benefits are undeniable. Clinical trials have explored several drugs and treatment strategies but none have resulted in significant improvement. Sadly, this is also true of later-approved drugs due to mechanisms of drug resistance. One of the most promising potential tumor growth inhibitors, Ferroptosis, can impact HCC development and progression by manipulating intracellular iron levels and ROS (180). Ferroptosis, however, is understudied in human clinical trials, and is mainly investigated on animal models. The evidence for its mechanisms at the molecular level are relatively limited.

Whether it is possible to clearly distinguish ferroptosis from other forms of PCD during pathogenesis, and to carry out targeted prevention and treatment, is also worthy of further investigation.

## 5 Conclusion and prospects

The liver functions as an immunoregulatory organ, containing a rich array of adaptive immune cells that can suppress inflammation to a certain extent (181, 182). The interactions within the TIME are intricate, dependent on the populations of immune cells present, and predictive of how well immunotherapies will function and how long patients will live. HCC is known to harbor TAMs, MDSCs, CAFs, TANs, TILs, DCs, and elements of the ECM within its TIME (183). Compared to other solid tumors, HCC faces a steep climb in effectively utilizing immunotherapy due to its immunosuppressive TIME. In HCC, nearly all cell subpopulations and an army of regulatory processes conspire to advance the tumor’s malignancy.

Macrophage research and advances in immunotherapy have provided some inroads into managing liver cancer, yet the road to fully tackling this disease remains beset with many challenges. Objective response rates can still be too low and adverse treatment effects occur with discouraging frequency. To surmount these obstacles and realize personalized precision treatment plans for each liver cancer patient, there is a clear need to take a bird’s eye view analysis, evaluate, and predict treatment outcomes, and explore new combination therapy techniques. Targeting liver-specific immune environment macrophages with more stable, safe, and effective immunotherapeutic methods, alongside traditional Chinese medicine, will further advance the treatment of liver cancer.
